# Osteosclerotic Metaphyseal Dysplasia Due to a Likely Pathogenic LRRK1 Variant as a Cause of Recurrent Long Bone Fractures

**DOI:** 10.1002/jbm4.10755

**Published:** 2023-06-28

**Authors:** Evert F.S. van Velsen, Serwet Demirdas, David Hanff, M. Carola Zillikens

**Affiliations:** ^1^ Department of Internal Medicine Erasmus Medical Center Rotterdam The Netherlands; ^2^ Erasmus MC Bone Center Erasmus Medical Center Rotterdam The Netherlands; ^3^ Department of Clinical Genetics Erasmus Medical Center Rotterdam The Netherlands; ^4^ Department of Radiology and Nuclear Medicine Erasmus Medical Center Rotterdam The Netherlands

**Keywords:** fractures, LRRK1, osteopetrosis, osteoporosis, osteosclerotic metaphyseal dysplasia

## Abstract

Osteosclerotic metaphyseal dysplasia (OSMD) is a very rare autosomal‐recessive disease caused by mutations in the leucine‐rich repeat kinase 1 (LRRK1) gene. It is a sclerosing skeletal dysplasia characterized by osteosclerosis of the long bones, predominantly at the metaphyses and vertebrae. Phenotypic features can be short stature, pathological fractures, delayed development, and hypotonia, but they are not uniformly present, and relatively few cases are known from the literature. A 40‐year‐old man was seen at our bone center because of nonspontaneous multiple peripheral low‐energy trauma fractures since puberty. He had no other complaints and his family history was negative. Except for a relatively short stature (167 cm; −1.5 SD), there were no abnormalities on examination, including laboratory tests. Initially, a suspicion was raised of osteogenesis imperfecta, but bone mineral density was high and X‐rays of the whole skeleton showed osteosclerosis of the metaphyses of long bones and vertebrae. Whole‐exome sequencing showed a homozygous, likely pathogenic, variant (American College of Medical Genetics and Genomics criteria class 4) in the LRRK1 gene, fitting the diagnosis of OSMD. In conclusion, we described a 40‐year‐old patient with osteosclerotic metaphyseal dysplasia caused by a homozygous variant in the LRRK1 gene, resulting in multiple fractures of the long bones without other features of the disease, adding to the phenotypic variation of OSMD. © 2023 The Authors. *JBMR Plus* published by Wiley Periodicals LLC on behalf of American Society for Bone and Mineral Research.

## Graphical abstract

We present a case of a 40‐year old patient with osteosclerotic metaphyseal dysplasia (OSMD; characterized by osteosclerosis of the long bones) caused by a homozygous variant in the LRRK1 gene, causing multiple fractures of the long bones and vertebral bodies, but no other features of the disease, such as delayed development, late‐onset paraplegia, seizures, and hypotonia, were present. Our study adds to the phenotypic variation of OSMD.

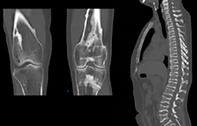

## Introduction

Osteosclerotic metaphyseal dysplasia (OSMD, Online Mendelian Inheritance in Man [OMIM] No. 615198) is a very rare autosomal‐recessive disease with a prevalence of <1:1.000.000 caused by mutations in the leucine‐rich repeat kinase 1 (LRRK1, OMIM No. 610986) gene.^(^ ^1^ ^)^ It is a sclerosing skeletal dysplasia characterized by osteosclerosis of the long bones (predominantly at the metahphyses) and vertebrae.^(^ ^2^ ^)^ Studies in knockout mice indicate that LRRK1‐deficient mice exhibit severe osteopetrosis, with defective osteoclasts that can only form superficial bone erosions (“pseudo‐resorption”).^(^ ^3^ ^)^ In humans, due to impaired bone degradation, bones become brittle, leading to pathologic fractures. Additional features, which are not uniformly present, include short stature, developmental delay, hypotonia, late‐onset paraplegia, and seizures.^(^ ^4^ ^)^ Laboratory findings may include elevated levels of alkaline phosphatase, but often no abnormalities are found.^(^ ^4^ ^)^ As only few patients with OSMD have been reported to date, with very different presentations,^(^ ^1^, ^4^, ^5^, ^6^, ^7^, ^8^ ^)^ the phenotypic spectrum of this rare skeletal dysplasia has yet to be fully elucidated. Additionally, without awareness, patients may be misclassified (e.g., as osteogenesis imperfecta [OI]) based on the presence of multiple fractures alone. Therefore, we present here a case of a 40‐year‐old man with OSMD having, besides multiple fractures, relatively few other problems.

## Patient Case

A 40‐year‐old man was seen for a second opinion at the Erasmus MC Bone Center because of multiple peripheral fractures since puberty. After a fracture of the right femur when he was 11 years old, he broke his left femur (once), his left tibia (twice), his right tibia (three times), his right ulna, and again his right femur (twice). These fractures usually occurred after falling, tripping, or contact during sports, mostly with minimal force. Because of the multiple fractures, a suspicion of a form of OI had been raised at some point.

As far as he could tell, he had a normal development, and no hearing, teeth, dermal, or gastrointestinal problems. He is married and has two children. There is no evident history of bone problems in his family; his mother broke her wrist after falling from a chair, and his brother broke his upper leg twice at the same place during kickboxing. His family originates from Turkey, and there is no known consanguinity. Except for cholecalciferol, he never used medication for osteoporosis.

At physical examination, a male fitting his age was seen with no apparent abnormalities or deformities. His height was 167 cm (−1.5 SD) with a weight of 70 kg and BMI of 25.1 kg/m^2^.

**Fig. 1:**
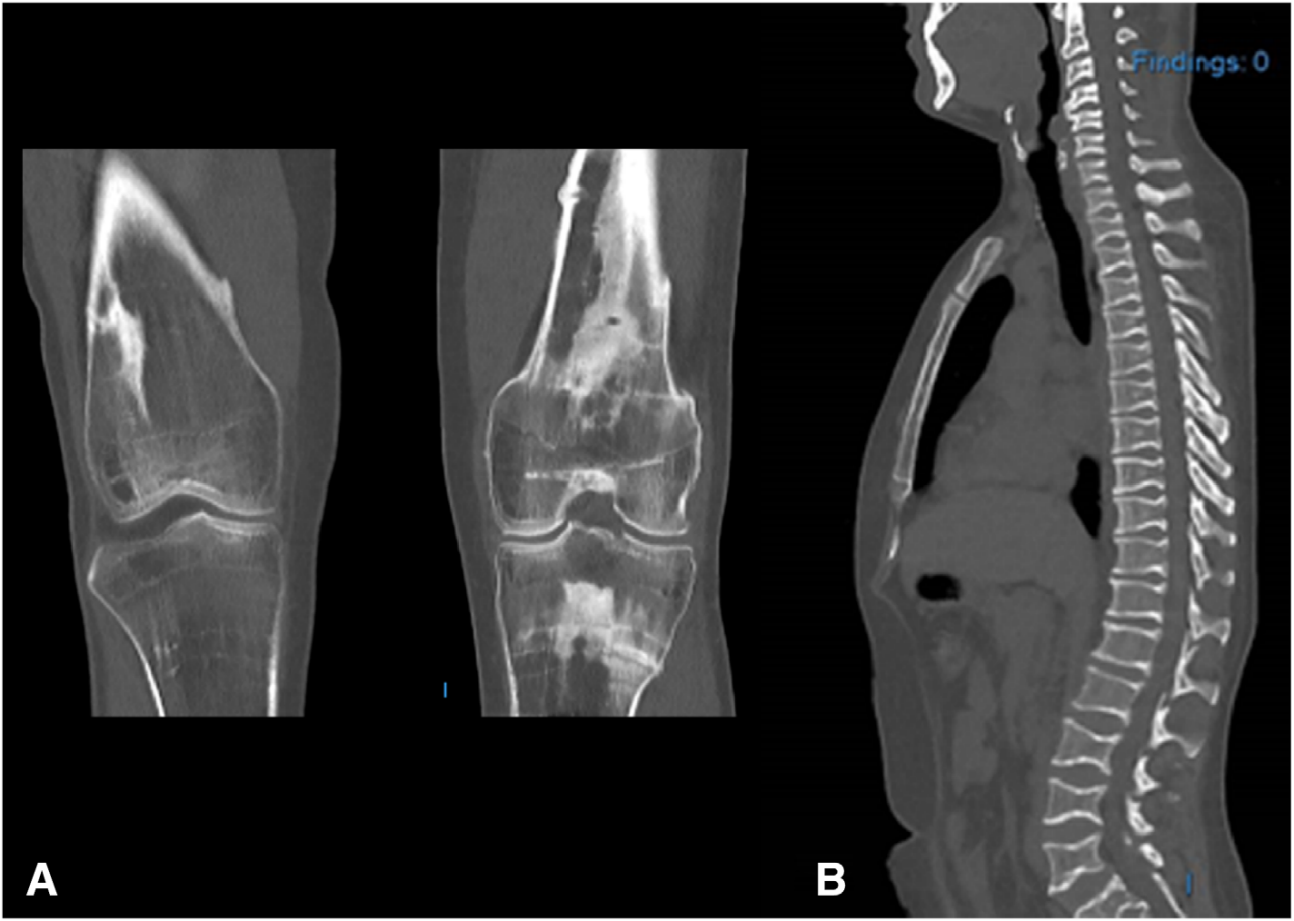
Typical radiologic characteristics fitting with osteosclerotic metaphyseal dysplasia of femur/tibiae (A) and vertebrae (B). (A) Coronal CT image of both knees showing bilateral, but predominantly on the left knee, sclerotic bands in the metaphysis of the femur and tibia in transverse and longitudinal direction. This resembles an osteopathia striata, but largely located in the metaphysis. (B) Sagittal CT image of the spine shows biconcave loss of height of the vertebrae with a fish‐like appearance. Some vertebrae also show more sclerotic endplate changes.

**Fig. 2:**
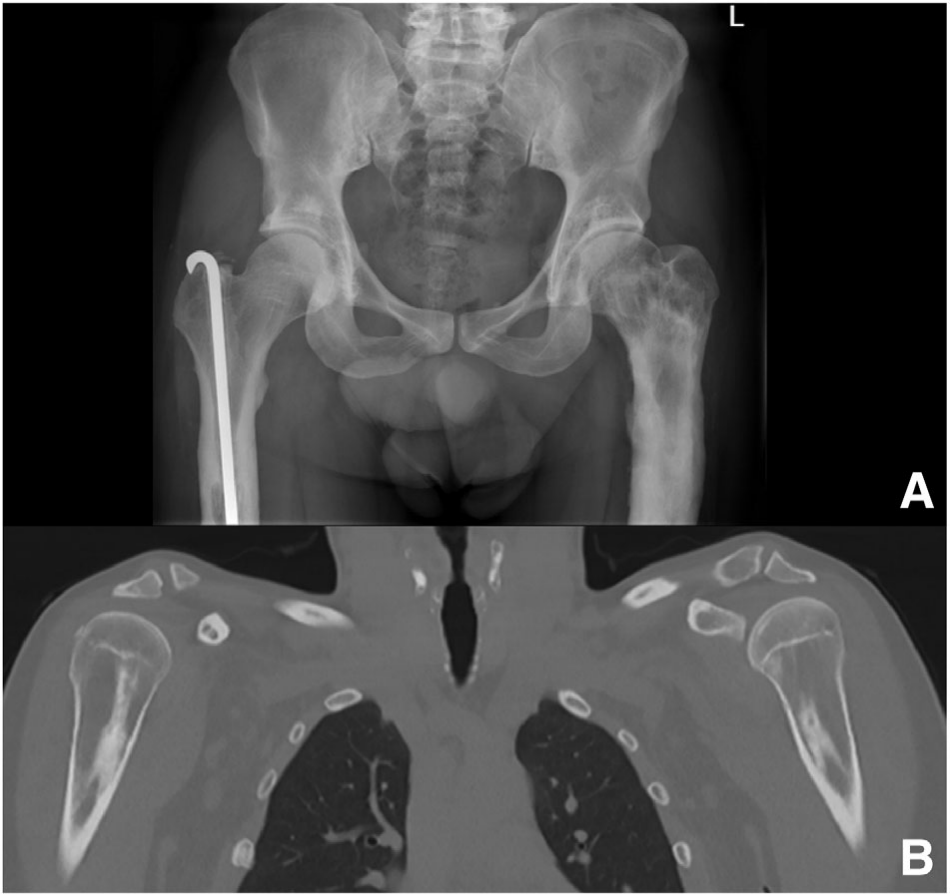
Typical radiologic characteristics fitting with osteosclerotic metaphyseal dysplasia of pelvis (A) and humeri (B). (A) AP radiograph of pelvis showing postoperative status on right with fixation nail centrally in proximal femur after fracture in history. Sclerotic bands in neck of femur and diffuse sclerotic changes in proximal femur diaphysis are evident, partly due to healed fractures. Focal sclerotic changes are bilateral in the acetabulum with “bone‐in‐bone” appearance. (B) Coronal image of humeri with sclerotic bands in metadiaphysis in longitudinal direction, just as in femur and tibia.

Blood tests showed no abnormalities, especially regarding calcium, phosphate, bone markers, and vitamin D (see also Table 1). Further, 24‐hour urine collection showed normal calcium excretion (3.45 mmol/24 h). A dual‐energy X‐ray absorptiometry scan showed an increased bone mineral density (BMD) of both the lumbar spine (LS), femur neck (FN), and total body (TB) (LS T + 4.9 SD; FN [left] T + 2.6 SD; TB T + 3.1 SD. LS Z + 4.9 SD; FN [left] Z + 2.9 SD; TB Z + 3.1 SD) suggestive of a primary bone dysplasia with increased BMD. Additional CT imaging was performed showing some characteristics of OSMD like sclerosis of the metaphysis and epiphyseal margins of the femura and tibiae and of the vertebrae (Figs. 1 and 2). Therefore, the patient was referred to our clinical geneticist to perform further analyses. Whole‐exome sequencing (WES) showed a homozygous, likely pathogenic, variant (class 4 per American College of Medical Genetics and Genomics criteria)^(^ ^9^ ^)^ in the LRRK1 gene (NM_024652.5[LRRK1]:c.5791delA, p. [Ile1931fs]). Mutations in the LRRK1 gene were earlier described in OSMD.^(^ ^5^, ^6^ ^)^ As currently, except for the recurring fractures, no other problems were observed from the disease, no interventions were needed, and the patient will be followed at our outpatient clinic.

**Table 1:** Laboratory values at diagnosis

	Reference range	Unit	At diagnosis
Serum			
Calcium	2.20–2.65	mmol/L	2.49
Phosphate	0.80–1.40	mmol/L	1.32
Creatinin	50–100	umol/L	65
PTH	0.68–4.40	pmol/L	1.5
Vitamin D 25 OH	50–120	nmol/L	53
Alkaline phosphatase	< 115	U/L	111
b‐CTx	< 0.58	μg/L	0.14
P1NP	19.4–95.4	ng/mL	23
Bone alkaline phosphatase	< 20.1	μg/L	15.2
ASAT	< 35	U/L	19
ALAT	< 45	U/L	14
24‐hour urine			
Creatinine	7.1–17.7	mmol/24 h	11.4
Calcium	2.5–7.5	mmol/24 h	3.45
Phosphate	13.0–42.0	mmol/24 h	31.8

## Discussion

OSMD is a very rare disease, first described by Nishimura and Kozlowski in 1993,^(^ ^2^ ^)^ and more than 20 years later the causal gene, LRRK1, was found.^(^ ^5^, ^6^ ^)^ Several cases have been described in the literature showing the multiple features of the disease.^(^ ^1^, ^4^, ^5^, ^6^, ^7^, ^8^, ^10^ ^)^ Next to the hallmark radiological feature of osteosclerosis of the long bones (predominantly at the metaphyses) and vertebrae, several additional phenotypic features have been described. These include pathologic fractures, short stature, developmental delay, hypotonia, late‐onset paraplegia, optic nerve atrophy, seizures, dental abnormalities, pulmonary problems, and osteonecrosis of the jaw.^(^ ^1^, ^4^, ^5^, ^6^, ^7^, ^8^ ^)^ However, these features are not present in all patients, and even those with the same mutation can harbor different features, indicating variable expressivity.^(^ ^4^ ^)^ Therefore, its full phenotypic spectrum has yet to be elucidated. Our patient, to the best of our knowledge the oldest described patient in the literature, suffered since childhood mainly from multiple fractures of the long bones of his legs, except for a recent ulna fracture, and has a relatively short stature (167 cm; SD −1.5). However, no other features of the earlier described phenotype were observed, although no hearing test was performed because of a lack of complaints, again indicating the variation of phenotypic features of the disease. The absence of other features had even led previously to a presumptive diagnosis of OI.

Studies in knockout mice indicate that LRRK1‐deficient mice exhibit severe osteopetrosis, with defective osteoclasts that can only form superficial bone erosions (“pseudo‐resorption”).^(^ ^1^, ^3^ ^)^ A bone biopsy from a patient with OSMD showed an increase in trabecular bone mass with flat‐shaped, multinucleated osteoclasts, without visible resorption lacunae indicative of defective bone resorption.^(^ ^1^ ^)^ These histopathological features are consistent with the reported normal osteoclastogenesis but abnormal osteoclast function exhibited in this disorder.^(^ ^3^ ^)^ In our patient, no bone biopsy was taken because the combination of clinical features and the discovered biallelic LRRK1 variant was sufficient to diagnose OSMD, and performing a bone biopsy would, currently, not have any clinical consequences.

The medical treatment for patients with osteopetrosis, and osteopetrosis‐like diseases such as OSMD, is an ongoing clinical problem. There are no effective and safe therapeutic approaches, but patient education about the disease and potential complications is of great importance. Hematopoietic stem cell transplantation (HSCT) is currently the only effective treatment for the lethal forms of osteopetrosis.^(^ ^4^, ^11^ ^)^ Today, the use of novel and optimized HSCT protocols has led to a greater rate of HSC engraftment, raising life expectancy of transplanted patients. Nevertheless, this procedure still carries several important side effects, and it is not always successful.^(^ ^11^ ^)^ Additionally, the procedure does not reverse existing damage to vital structures. The recurring fractures in our patient are not severe enough to warrant HSCT, but currently, as far as we know, there exists no effective medication in terms of preventing new fractures. Further research is therefore needed to fill this knowledge gap.

In conclusion, we presented the history of a 40‐year‐old patient with the very rare skeletal bone dysplasia OSMD caused by a homozygous variant in the LRRK1 gene causing multiple fractures of the long bones without other phenotypic features of the disease. This case adds to the known phenotypic variation of the disease and may also create awareness of rare causes of bone fragility.

## Author Contributions

**Evert F.S. van Velsen:** Conceptualization; data curation; investigation; visualization; writing – original draft; writing – review and editing. **Serwet Demirdas:** Conceptualization; data curation; investigation; writing – review and editing. **David Hanff:** Conceptualization; data curation; investigation; visualization; writing – review and editing. **M. Carola Zillikens:** Conceptualization; data curation; investigation; supervision; writing – review and editing.

## Conflicts of Interest

EVV, SD, DH, and MCZ declare that no conflicts of interest and no competing financial interests exist.

## Funding Information

This research did not receive any specific grant from any funding agency in the public, commercial, or not‐for‐profit sector.

## Patient Consent Statement

The described patient gave written informed consent for publication of this manuscript.

### Peer Review

The peer review history for this article is available at https://www.webofscience.com/api/gateway/wos/peer-review/10.1002/jbm4.10755.
